# Mediating role of loneliness and emotional disturbance in the association between childhood trauma and occupational burnout among nurses: a cross-sectional study

**DOI:** 10.3389/fpsyt.2024.1394289

**Published:** 2024-05-17

**Authors:** Jing Hu, Mengxi Zhai, Donghui Fu, Zhizhou Duan, Xiangfan Chen

**Affiliations:** ^1^ Department of Cardiovascular, Jiangxi Provincial People’s Hospital, The First Affiliated Hospital of Nanchang Medical College, Nanchang, Jiangxi, China; ^2^ School of Public Health, Wuhan University, Wuhan, Hubei, China; ^3^ Department of Urology, Jiangxi Provincial People’s Hospital, The First Affiliated Hospital of Nanchang Medical College, Nanchang, Jiangxi, China; ^4^ Preventive Health Service, Jiangxi Provincial People’s Hospital, The First Affiliated Hospital of Nanchang Medical College, Nanchang, Jiangxi, China; ^5^ Department of Biobank, Nantong First People’s Hospital, Nantong, Jiangsu, China

**Keywords:** nurse, childhood trauma, loneliness, emotional disturbance, occupational burnout

## Abstract

**Background:**

The occupational burnout status of nurses in China warrants attention. Childhood trauma, loneliness, and emotional disturbance are significant predictors of this burnout, yet few studies have delved into the underlying mechanisms. This study seeks to explore the mediating pathway from childhood trauma to loneliness, emotional disturbance, and ultimately occupational burnout among nurses through a cross-sectional analysis.

**Method:**

Data for the study were collected from Yunnan province, China, from 11 July to 26 July 2022. Key variables were measured using standardized scales: the Childhood Trauma Questionnaire Short Form for childhood trauma, the three-item loneliness scale for loneliness, the Patient Health Questionnaire and the Generalized Anxiety Disorder questionnaire for emotional disturbance, and the Maslach Burnout Inventory-Human Service Survey for occupational burnout. Mediation modeling analysis was employed for data analysis to test the effect of loneliness and emotional disturbance on the association between childhood trauma and occupational burnout. Data analysis was conducted using AMOS and SPSS software.

**Results:**

Loneliness and emotional disturbance fully mediated the association between childhood trauma and emotional exhaustion [indirect effect (95% CI) = 0.228 (0.196, 0.270)]. Loneliness and emotional disturbance partially mediated the association between childhood trauma [indirect effect (95% CI) = −0.020 (−0.039, 0.002)] and personal accomplishment or depersonalization [indirect effect (95% CI) = 0.221 (0.186, 0.255)].

**Conclusion:**

Childhood trauma could affect occupational burnout through loneliness and emotional disturbance among nurses. Preventive strategies could include protective interventions like treatment of loneliness and emotional disturbance, especially in nurses who experienced childhood trauma.

## Introduction

### Greater risk of occupational burnout among nurses

Occupational burnout, recognized as a chronic work-related stress problem by the World Health Organization in its International Classification of Diseases, is a common syndrome among practitioners. While it tends to occur in workers across various industries, medical and health workers, including nurses, are particularly susceptible (1, 2), Nurses, because of their direct and constant interaction with patients and families, coupled with high-intensity and complex nursing tasks, often experience higher levels of burnout compared to other medical professionals (3). A systematic review revealed that 28% of nurses experienced high emotional exhaustion, 15% experienced high depersonalization, and 31% reported low personal accomplishment (4). Despite this, research on nurse burnout remains limited in China, despite the significant stressors nurses face due to the country’s large population and limited medical resources (5). With an increasing number of medical incidents adding to their burdens, nurses in China face significant physical and mental strain. Thus, the occupational burnout status of nurses warrants attention in China.

### Effect of childhood trauma on occupational burnout

Childhood trauma refers to a variety of potentially persistent stressful or traumatic events that individuals experience physically, psychologically, and emotionally before the age of 16 (6). According to the life course theory, certain experiences or events can serve as turning points in an individual’s life trajectory, particularly when they occur during childhood or adolescence, significantly impacting the subsequent course of one’s life (7). Therefore, we hypothesized that nurses’ childhood traumatic experiences may impact their occupational burnout and different types of occupational burnout. However, there are few studies exploring this, indicating the need for further investigation.

### Potential mediation mechanism between childhood trauma, loneliness, emotional disturbance, and occupational burnout

The stress sensitivity theory posits that individuals who have experienced childhood trauma have lower stress thresholds and are more prone to experiencing adverse mental health outcomes, such as loneliness and depressive symptoms, compared to those who have not experienced childhood trauma (8). Studies (9, 10) have indicated that exposure to negative events in childhood, such as abuse and neglect, can elevate the risk of loneliness and depressive symptoms. Hence, it is plausible that nurses who have experienced more childhood trauma may be predisposed to developing loneliness and depressive symptoms. However, research on this relationship among nurses is scarce, leaving the effects of childhood trauma on loneliness, depressive symptoms, and occupational burnout uncertain.

Moreover, it is widely acknowledged that poor mental health, including loneliness and depressive symptoms, can heighten the risk of occupational burnout among nurses, encompassing emotional exhaustion and depersonalization (11, 12). Additionally, poor mental health may diminish nurses’ sense of personal accomplishment (13). According to the affective event theory, external encounters experienced by nurses can influence their emotional state and subsequently impact their work status and behavior (14, 15). A study conducted in Brazil found that nurses with depressive symptoms were 5.33 times more likely to experience occupational burnout (16), while a study in Chile revealed that nurses experiencing loneliness were less likely to achieve personal accomplishment (17). Therefore, it is conceivable that loneliness and emotional disturbance may mediate the relationship between childhood trauma and burnout among nurses.

In addition, numerous studies have underscored the profound association between loneliness and emotional disturbances, particularly anxiety and depressive symptoms. For instance, Creese et al. (2021) identified loneliness as a significant risk factor for anxiety and depression among the elderly, both before and after the COVID-19 pandemic (18). Furthermore, a 5-year longitudinal study conducted in Chicago revealed that loneliness serves as a predictor for subsequent escalation in depressive symptoms (19).

### Purpose of the study

The purpose of this study is to investigate the chained mediation mechanism from childhood trauma to loneliness, depressive symptoms, and further to occupational burnout (including emotional disturbance, depersonalization, and overall burnout) among nurses in China. The ultimate goal is to deepen our understanding of the underlying mechanisms of occupational burnout and provide valuable insights to enhance the efficiency and effectiveness of nursing practices.

## Method

### Participants and procedure

Data for this study were derived from a cross-sectional survey of nurses in Yunnan province, China. The participants were nurses working at 18 local governmental hospitals, excluding student nurses, who agreed to provide informed consent and were able to understand and complete the questionnaire. Recruitment took place in the Dehong districts of Yunnan province. Dehong, situated in southwest China, shares borders with Myanmar on its north, west, and south sides.

Participants were recruited using convenience sampling. An electronic questionnaire was distributed to nurses via the “Wenjuanxing” online platform for data collection. Trained researchers briefed each participant on the study, and the questionnaire link was distributed with the assistance of the nursing departments of each governmental hospital. The survey ensured anonymity and independence in responses, with any queries addressed by the investigators, and participants had the right to withdraw at any time. Ultimately, 1,965 nurses participated in the survey, with 90.3% (1,774) completing it. This study received approval from the Ethics Committee of Dehong People’s Hospital in China (Number: DYLL-KY032).

### Socio-demographic variables

The demographic characteristics included age (classified in years), sex (male and female), ethnicity (Han and others), legal residence (urban and rural), education level (high school or lower and bachelor’s degree or above), marital status (married, unmarried, and divorced or other), monthly income (3,000 RMB or lower, 3,001–5,000 RMB, 5,001–7,000 RMB, and 7,000 RMB or higher), and work experience (classified in years).

### Childhood trauma

Childhood trauma was measured by the Childhood Trauma Questinnaire Short Form (CTQ-SF) (6), which inquired about participants’ experiences before the age of 16. The scale includes items related to emotional abuse, physical abuse, sexual abuse, emotional neglect, and physical neglect. The CTQ-SF comprises 25 clinical items and three validity items. Each item (e.g., “I feel that someone in my family hates me”) is rated on a five-point Likert scale ranging from 1 (never) to 5 (always), with the total score ranging from 25 to 125. The Cronbach’s α coefficient for internal consistency in this study was 0.73.

### Loneliness

The three-item loneliness scale was used to measure loneliness (20). Each item (e.g., “How often do you feel that you lack companionship?”) is rated on a three-point Likert scale ranging from 1 (hardly ever) to 3 (often), with the total score ranging from 3 to 9. Higher scores indicate a higher level of loneliness. The Cronbach’s α coefficient for internal consistency in this study was 0.83.

### Emotional disturbance

The nine-item Patient Health Questionnaire (PHQ-9) (21) was utilized to measure depressive symptoms. Each item is assessed using a four-point Likert scale ranging from 0 (not at all) to 3 (nearly every day), with the total score ranging from 0 to 27. A higher score indicates more severe depressive symptoms. The Cronbach’s α coefficient for internal consistency in this study was 0.91.

The generalized anxiety disorder-7 (GAD-7) (22) was employed to measure anxiety symptoms. Each item is assessed using a four-point Likert scale ranging from 0 (not at all) to 3 (nearly every day), with the total score ranging from 0 to 21. A higher score indicates more severe anxiety symptoms. The Cronbach’s α coefficient in this study was 0.93.

### Occupational burnout

The Chinese version of the Maslach Burnout Inventory-Human Service Survey (MBI-HSS) (23, 24), which comprises three subscales, was used to measure occupational burnout among nurses. This study was granted permission by the Mind Garden company. The three subscales include emotional exhaustion (Cronbach’s alpha = 0.92, nine items, e.g., “I feel emotionally drained from my work”), personal accomplishment (Cronbach’s alpha = 0.83, eight items, e.g., “I have accomplished many worthwhile things in this job”), and depersonalization (Cronbach’s alpha = 0.87, five items, e.g., “I feel treated some recipients as if they were impersonal objects”). Each item is assessed using a seven-point Likert scale ranging from 0 (not at all) to 6 (nearly every day). The scores of the items in each subscale are summed to obtain the total score for each dimension.

### Statistical analysis

Descriptive analyses (e.g., frequency, proportion, mean, and standard deviation) were employed to describe the sample characteristics. Pearson correlation was conducted to explore the relationships among childhood trauma, loneliness, emotional disturbance (depressive symptoms and anxiety symptoms), and the dimensions of MBI-HSS (emotional exhaustion, personal accomplishment, and depersonalization).

**Figure 1:**
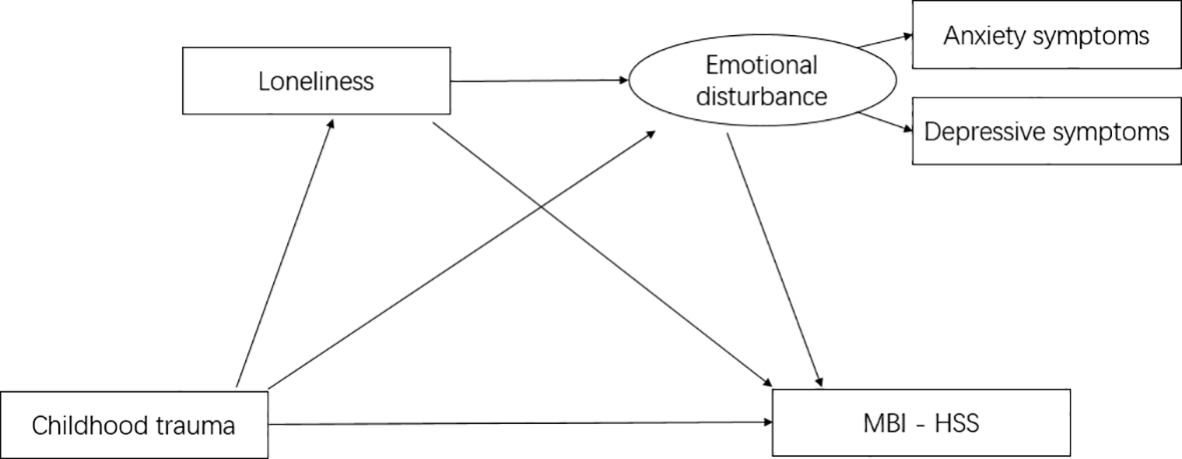
The conceptual model for the whole sample, based on a previous study.

Mediation analysis was utilized to examine the mediating role of loneliness and emotional disturbance in the association between childhood trauma and the various dimensions of MBI-HSS (25, 26). **Figure 1** illustrates the conceptual diagrams of the mediation model. A total of 3,000 bootstrap cycles were performed to compute the standardized total effect and indirect effect, along with standard errors and bias-corrected 95% confidence intervals. Indirect effects were calculated as the total effect minus the direct effect. All data analyses were conducted using AMOS version 24.0 and SPSS version 24.0 (IBM Inc., NY, USA).

## Results

### Characteristics of the study sample

**Table 1** presents the socio-demographic characteristics and key variable outcomes of the participants. The mean age of the participants was 32.00 (SD = 7.99) years. The majority were women (93.9%), with over half identifying as Han and married. Nearly 65% had attained a bachelor’s degree or higher, and over half (77.60%) resided in rural areas. The mean scores for childhood trauma and loneliness were 36.86 (SD = 10.73) and 5.26 (SD = 1.55), respectively. The mean scores for depressive symptoms and anxiety symptoms were 7.42 (SD = 5.13) and 6.29 (SD = 4.32). Additionally, the mean scores for emotional exhaustion, personal accomplishment, and depersonalization were 20.19 (SD = 12.50), 28.61 (SD = 11.50), and 6.88 (SD = 6.57), respectively.

**Table 1:** Socio-demographic characteristics and key variable outcomes of participants (*N* = 1,774).

Characteristic	Number	Percent (%)
Age (years)	32.00 ± 7.99	
20–24	185	10.4
25–29	674	38.0
30–34	452	25.5
35–39	171	9.6
40–59	292	16.5
Sex		
Women	1,666	93.9
Men	108	6.1
Ethnic		
Han	1,276	71.9
Others	498	28.1
Marital status		
Unmarried	517	29.1
Married	1,200	67.6
Divorce/others	57	3.2
Residence		
Rural	1,071	60.4
Urban	703	39.6
Education level		
High school or lower	614	34.6
Bachelor’s degree or above	1,160	65.4
Income (monthly)		
3,000 or lower	498	28.1
3,001–5,000	782	44.1
5,001–7,000	325	18.3
7,000 or higher	169	9.5
Experience (years)	10.83 ± 8.55	
0–4	393	22.2
5–9	592	33.4
10–14	391	22.0
15–19	128	7.2
20–40	270	15.2
Key variables (mean ± SD)		
Childhood trauma	36.86 ± 10.73	
Loneliness	5.26 ± 1.55	
Depressive symptoms	7.42 ± 5.13	
Anxiety symptoms	6.29 ± 4.32	
MBI-HSS Emotional exhaustion	20.29 ± 12.50	
MBI-HSS Personal accomplishment	28.61 ± 11.50	
MBI-HSS Depersonalization	6.88 ± 6.57	

### Correlation coefficient of key variables using Pearson correlation among participants

Results in **Table 2** indicate that childhood trauma was positively correlated with loneliness (*r* = 0.290, *p* < 0.001), depressive symptoms (*r* = 0.290, *p* < 0.001), and anxiety symptoms (*r* = 0.271, *p* < 0.001). Among different types of occupational burnout, emotional exhaustion was positively correlated with childhood trauma (*r* = 0.198, *p* < 0.001), loneliness (*r* = 0.453, *p* < 0.001), depressive symptoms (*r* = 0.621, *p* < 0.001), and anxiety symptoms (*r* = 0.580, *p* < 0.001). Personal accomplishment was negatively correlated with childhood trauma (*r* = −0.229, *p* < 0.001), loneliness (*r* = −0.107, *p* < 0.001), depressive symptoms (*r* = −0.141, *p* < 0.001), and anxiety symptoms (*r* = −0.153, *p* < 0.001). Depersonalization was positively correlated with childhood trauma (*r* = 0.317, *p* < 0.001), loneliness (*r* = 0.366, *p* < 0.001), depressive symptoms (*r* = 0.510, *p* < 0.001), and anxiety symptoms (*r* = 0.463, *p* < 0.001).

**Table 2:** Correlation coefficient of key variables using Pearson correlation.

Variables	1	2	3	4	5	6	7
1. Childhood trauma	1						
2. Loneliness	0.290^***^	1					
3. Depressive symptoms	0.290^***^	0.487^***^	1				
4. Anxiety symptoms	0.271^***^	0.482^***^	0.807^**^	1			
5. MBI-HSS Emotional exhaustion	0.198^***^	0.453^***^	0.621^***^	0.580^***^	1		
6. MBI-HSS Personal accomplishment	−0.299^***^	−0.107^***^	−0.141^***^	−0.153^***^	0.992	1	
7. MBI-HSS Depersonalization	0.317^***^	0.366^***^	0.510^***^	0.463^***^	0.746^***^	−0.054^*^	1

^***^p < 0.001.

### Mediation mechanism between childhood trauma, loneliness, emotional disturbance, and occupational burnout

**Figure 2:**
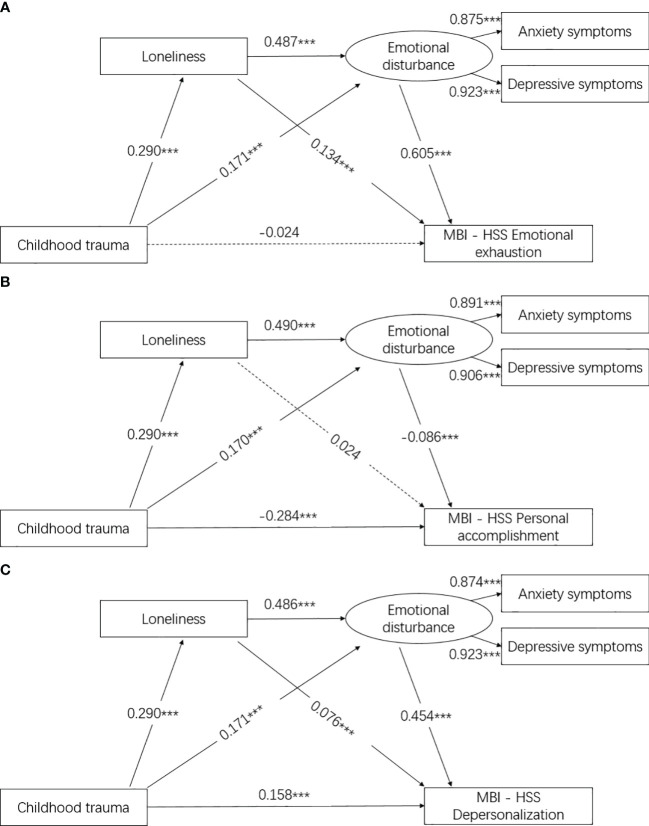
**(A)** Association between childhood trauma, loneliness, emotional disturbance, and MBI-HSS emotional exhaustion. model fit: CMIN/DF = 3.688, NFI = 0.990, CFI = 0.993, TLI = 0.985, RFI = 0.979, RMSEA = 0.039, SRMR = 0.024; results were shown as the standardized β value, and sex and age were adjusted in the models; ^***^ was represented for *p* < 0.001. **(B)** Association between childhood trauma, loneliness, emotional disturbance, and MBI-HSS personal accomplishment. model fit: CMIN/DF = 3.529, NFI = 0.988, CFI = 0.991, TLI = 0.981, RFI = 0.974, RMSEA = 0.038, SRMR = 0.023; results were shown as the standardized β value, and sex and age were adjusted in the models; ^***^ was represented for *p* < 0.001. **(C)** Association between childhood trauma, loneliness, emotional disturbance, and MBI-HSS depersonalization. model fit: CMIN/DF = 3.870, NFI = 0.988, CFI = 0.991, TLI = 0.982, RFI = 0.976, RMSEA = 0.040, SRMR = 0.023; results were shown as the standardized β value, and sex and age were adjusted in the models; ^***^ was represented for *p* < 0.001.

Results in **Table 3** and **Figure 2A** reveal that loneliness [indirect effect (95% CI) = 0.294 (0.262, 0.328)] and emotional disturbance fully mediated the association between childhood trauma [indirect effect (95% CI) = 0.228 (0.196, 0.270)] and emotional exhaustion. Similarly, results in **Table 3** and **Figure 2B** indicate that loneliness [indirect effect (95% CI) = −0.042 (−0.072, −0.008)] and emotional disturbance partially mediated the association between childhood trauma [indirect effect (95% CI) = −0.020 (−0.039, 0.002)] and personal accomplishment. Moreover, results in **Table 3** and **Figure 2C** demonstrate that loneliness [indirect effect (95% CI) = 0.221 (0.186, 0.255)] and emotional disturbance partially mediated the association between childhood trauma [indirect effect (95% CI) = 0.164 (0.132, 0.193)] and depersonalization. All models exhibit a good fit for the mediation modeling analysis. Additionally, the subscales (emotional abuse, physical abuse, sexual abuse, emotional neglect, and physical neglect) of CTQ-SF affect loneliness, emotional disturbance, and burnout. Please refer to **Supplementary Table 1** for details.

**Table 3:** Standardized total effect and indirect effect of study variables on MBI-HSS.

Variables	MBI-HSS Emotional exhaustion		MBI-HSS Personal accomplishment		MBI-HSS Depersonalization	
	Indirect effect β (95% CI)	Total effect β (95% CI)	Indirect effect β (95% CI)	Total effect β (95% CI)	Indirect effect β (95% CI)	Total effect β (95% CI)
Childhood trauma	0.228 (0.196, 0.270)	0.204 (0.152, 0.250)	−0.020 (−0.039, 0.002)	−0.304 (−0.346, −0.260)	0.164 (0.132, 0.193)	0.322 (0.273, 0.370)
Loneliness	0.294 (0.262, 0.328)	0.428 (0.380, 0.464)	−0.042 (−0.072, −0.008)	−0.019 (−0.079, 0.035)	0.221 (0.186, 0.255)	0.297 (0.244, 0.343)
Emotional disturbance	–	0.605 (0.560, 0.650)	–	−0.086 (−0.141, −0.019)		0.454 (0.378, 0.501)

## Discussion

Occupational burnout among medical staff, including nurses, has been a significant public health issue in China. This study aims to explore the underlying mediation mechanisms between childhood trauma, loneliness, emotional disturbance, and different categories of occupational burnout among nurses. It holds great significance for formulating and implementing effective intervention and prevention programs for nurse burnout.

The findings of the study indicated high scores of emotional exhaustion and depersonalization, along with low levels of personal accomplishment among Chinese nurses. Previous studies on nurses’ occupational burnout have also reported similar trends (27, 28). Disparities in the level of occupational burnout are mainly attributed to differences in participants’ regions or ages. Therefore, psychological counseling and health education aimed at preventing job burnout should be prioritized in nurses’ continuing education and mental health initiatives.

Our study found that nurses with more childhood trauma were more likely to have high-level emotional exhaustion, depersonalization, and low-level personal accomplishment. The current research often ignores the childhood trauma of nurses themselves, and often focuses on the stress of nurses at work (26, 29, 30). The Life Course Theory (7) suggests that childhood is an important period of one’s life development, such that negative events that happened during this period will bring substantial undesirable effects on the individual’s entire life. Therefore, it is important to enhance the assessment of the mental health status of the nursing population, particularly regarding their childhood experiences. One approach to achieve this is by utilizing the Childhood Trauma Scale to assess nurses before they enter the profession. Nurses who have experienced childhood trauma should receive increased attention and psychological support.

In this study, we found that in addition to emotional disturbance, childhood trauma had a direct effect on both personal accomplishment and depersonalization. Emotional disturbance is characterized by emotional fatigue resulting from continual stress and inadequate stress management (31). This means that only persistent stress can lead to emotional exhaustion, whereas childhood trauma occurs before the age of 18 and cannot be counted as persistent stress, which may be why childhood trauma has no direct effect.

The underlying mechanisms between childhood trauma and job burnout are complex. The findings of this study indicated that loneliness and emotional disturbance may mediate the relationship. Nurses with childhood trauma may have more loneliness and emotional disturbance, increasing the risk of job burnout, including increasing the risk of emotional disturbance and depersonalization and reducing the level of personal accomplishment. Many studies (25, 32) have verified that loneliness can increase depressive symptoms in different populations. Previous studies have found that poor mental health could increase the risk of occupational burnout (including emotional exhaustion and depersonalization) among nurses (11, 33) and was more likely to decrease the level of personal accomplishment (33, 34). This is consistent with our research. Thus, in addition to increased care for nurses with childhood trauma, timely management and treatment of loneliness and emotional disturbance may be of great help for nurses. The establishment of specialized psychological counseling centers in hospitals and the development of appropriate health education may be effective in improving the mental health of nurses.

The study has several limitations. Firstly, it adopted a cross-sectional design, precluding the establishment of causal relationships. Secondly, the participants were exclusively from Yunnan province, China, which necessitates caution when generalizing findings to other regions. Thirdly, it did not include post-traumatic stress disorder (PTSD) as a variable, despite its known association with childhood trauma. PTSD is a significant outcome of traumatic events and could potentially mediate the relationship between childhood trauma and loneliness or emotional disturbance. Future studies should consider incorporating PTSD to obtain a more comprehensive understanding of the long-term psychological consequences of childhood trauma. Finally, childhood trauma, loneliness, emotional disturbance, and occupational burnout in this study were all self-reported, potentially introducing reporting bias.

## Conclusion

Our findings offer prospective evidence that childhood trauma could influence occupational burnout through loneliness and emotional disturbance among nurses. Future studies should give greater consideration to childhood trauma in the context of occupational burnout among nurses. Preventive strategies could involve protective interventions such as addressing loneliness and emotional disturbance, particularly in nurses who have experienced childhood trauma.

## Data availability statement

The original contributions presented in the study are included in the article/**Supplementary Material**. Further inquiries can be directed to the corresponding author.

## Ethics statement

The studies involving humans were approved by the Ethics Committee of Dehong People’s Hospital in China (Number: DYLL-KY032). The studies were conducted in accordance with the local legislation and institutional requirements. The participants provided their written informed consent to participate in this study.

## Author contributions

JH: Writing – original draft, Writing – review & editing. MZ: Writing – original draft. DF: Writing – original draft. ZD: Investigation, Writing – original draft, Writing – review & editing. XC: Writing – original draft.
