# Supplementary material for: Mediating role of loneliness and emotional disturbance in the association between childhood trauma and occupational burnout among nurses: a cross-sectional study
Source: Front Psychiatry. 2024 May 17;15:1394289. doi: 10.3389/fpsyt.2024.1394289 (PMC11140593; doi:10.3389/fpsyt.2024.1394289)
Supplement: Supplementary file 1 [file Table_1.docx]

| SEM model | Emotional abuse | Physical abuse | Sexual abuse | Emotional neglect | Physical neglect |
| --- | --- | --- | --- | --- | --- |
| Loneliness | 0.211*** | 0.110*** | 0.100*** | 0.302*** | 0.260*** |
| Emotional disturbance | 0.096*** | 0.100*** | 0.550*** | 0.471*** | 0.111*** |
| MBI-HSS emotional exhaustion | -0.028 | 0.190 | -0.014 | 0.014 | 0.006 |
| Each model shown good model fit and sex, and age were adjusted | | | | | |
| Loneliness | 0.302*** | 0.110*** | 0.100*** | 0.2111*** | 0.260*** |
| Emotional disturbance | 0.475*** | 0.103*** | 0.116*** | 0.095*** | 0.109*** |
| MBI-HSS personal accomplishment | -0.023 | -0.052* | -0.051* | -0.345*** | -0.235*** |
| Each model shown good model fit and sex, and age were adjusted | | | | | |
| Loneliness | 0.302*** | 0.110*** | 0.100*** | 0.260*** | 0.211*** |
| Emotional disturbance | 0.213*** | 0.100*** | 0.111*** | 0.111*** | 0.096*** |
| MBI-HSS depersonalization | 0.066** | 0.098*** | 0.103*** | 0.148*** | 0.110*** |
| Each model shown good model fit and sex, and age were adjusted | | | | | |

Table 1: sub scale (emotional abuse, physical abuse, sexual abuse, emotional neglect and physical neglect) of CTQ-SF affect loneliness, emotional disturbance, and burn out
